# Applicability of tactile memory examination as an option to visual- and verbal-based batteries

**DOI:** 10.1590/1980-57642021dn15-030010

**Published:** 2021

**Authors:** Omar Gurrola Arambula, Flavia Helena Pereira Padovani, Jose Eduardo Corrente, Andreas Batista Schelp, Felipe Jacques Sanches, Rogerio Martins Amorim, Arthur Oscar Schelp

**Affiliations:** 1 Department of Neurology, Psychology and Psychiatry, Faculdade de Medicina de Botucatu, Universidade Estadual Paulista “Júlio de Mesquita Filho” – Botucatu, SP, Brazil.; 2 Statistical Department, Bioscience Institute, Faculdade de Medicina de Botucatu, Universidade Estadual Paulista “Júlio de Mesquita Filho” – Botucatu, SP, Brazil.; 3 Hospital do Servidor Público São Paulo – São Paulo, SP, Brazil.; 4 Veterinary Clinical Department, Faculdade de Medicina Veterinária e Ciência Animal, Universidade Estadual Paulista “Júlio de Mesquita Filho” – Botucatu, SP, Brazil.

**Keywords:** memory, Alzheimer’s disease, tactile sense, neurocognitive test, memória, doença de Alzheimer, sentido tátil, teste neurocognitive

## Abstract

The validity and applicability of tactile battery tests for the diagnosis and medical follow-up of patients with Alzheimer’s disease and other dementia syndromes do not have their usefulness well understood in clinical practice. While haptic abilities in older individuals receive less attention, in earlier stages of human life they are well focused on. There are even fewer studies on tactile memory, including episodic memory of demented individuals with or without sensorial limitations. The applicability of a new haptic memory battery was evaluated in patients with Alzheimer’s disease with mild or moderate commitment.

**Objective:**

The aim of this study is to apply a battery based on tactile perception, recognition, and recollection of everyday objects in patients with Alzheimer’s disease, testing tactile delayed recall memory discrimination and late recognition to compare validated visual and verbal tests.

**Methods:**

Tactile-, visual-, and verbal-based memory performance was registered in 21 patients diagnosed with Alzheimer’s disease.

**Results:**

Except for tactile identification, it showed that there was a close relationship between the three sensory modalities of memory, with an apparent better performance of tactile incidental memory and recognition compared with the test with pictures.

**Conclusions:**

The haptic evaluation of memory demonstrated applicability in the evaluation of memory dysfunction in patients with Alzheimer’s disease. Further studies are needed to establish the sensibility and specificity of the proposed test that had a small sample size and many limitations.

## RESUMO

A aplicação de baterias baseadas em informações táteis para diagnóstico e acompanhamento de pacientes com doença de Alzheimer e outras demências não é muito difundida na prática clínica. Ao passo que a capacidade de reconhecimento tátil de objetos em crianças recebe bastante atenção dos pesquisadores, o mesmo não ocorre com indivíduos mais idosos. Existem ainda menos estudos abordando avaliação de memória episódica em pacientes idosos, com ou sem limitações sensoriais, como cegueira e surdez. O presente estudo propõe avaliar aplicabilidade de teste para avaliação de memória tátil em paciente com doença de Alzheimer.

**Objetivo:**

Aplicar em pacientes com doença de Alzheimer uma bateria baseada na percepção e reconhecimento táctil de objetos de uso cotidiano, avaliando a memória e reconhecimento tardio, comparado com testes visuais e verbais validados.

**Métodos:**

Foi avaliado o desempenho de baterias baseadas em informação táteis, visuais e verbais em 21 pacientes diagnosticados com a doença de Alzheimer.

**Resultados:**

Com a exceção da percepção tátil, foi demonstrada uma boa correlação entre as três modalidades de apresentação das informações a serem resgatadas tardiamente. Houve um desempenho aparentemente melhor do reconhecimento tátil quando comparado com apresentação de figuras impressas.

**Conclusões:**

A determinação da memória tátil em pacientes com doença de Alzheimer demonstrou ser aplicável como instrumento de avaliação dos distúrbios de memória presentes em portadores de doença de Alzheimer com comprometimento leve a moderado. As limitações do estudo, com reduzido tamanho da amostra, aponta para a necessidade de novos estudos com a devida validação, incluindo determinação de sensibilidade e especificidade, assim como pareamento, de acordo com sexo e idade com grupo controle.

## INTRODUCTION

The tests usually applied for the screening of cognitive impairment are visual-, verbal-, or paper-and-pencil-based tests.^1^ ^,^ ^2^

Sensory memory (i.e., a fraction of a second) is specific to the stimulus modality of presentation such as the acoustical echoic and iconic visual memories, and it must be distinguished from the short-term memory (i.e., usually up to 30 sec) and the long-term memory (i.e., declarative memory).^3^ Tactile perception is the mental process of becoming aware of or recognizing an object or idea and can be complemented by the haptic object recognition related to the choice of manual manipulation and exploratory strategies. The abilities are integrated as tactile agnosia functional system.

The studies of the short-term tactile memory perception compared with visual memory showed a lower tactile–spatial memory span than a visual–spatial span.^4^ Similarly, a study of working haptic memory perception of 2D images suggested a smaller memory capacity during tactile exploration.^5^ The author of that study suggested that the tactile system is almost amnestic when touch is perceived outside the fingertips. The presence of haptic manual habituation and discrimination of shape information in full-term newborns has already been well described.^6^ Of note, haptic recognition abilities disappear with interference from other sources, indicating that haptic memory is fragile at birth.^7^ On the other side of the lifespan, there are few reports on the subject^8^ and even fewer reports of tactile memory dysfunction in older individuals affected by amnestic degenerative diseases. An evaluation of angle discrimination comparing normal controls to patients with Alzheimer’s disease (AD) with mild cognitive impairment found that accuracy significantly decreased in the latter group.^9^ In a study with the assessment of dementia with a tactile battery, it was shown that demented patients performed significantly worse than controls, with a good correlation with Mini-Mental State Examination (MMSE) scores, in which the requested information was basically verbal.^10^ A visual and tactile combined evaluation of familial objects [i.e., Fuld Object-Memory Test (FOMT)] registered a moderate sensitivity to incipient dementia and a fair specificity as a predictor of dementia in cognitively normal elderly patients.^11^ ^,^ ^12^ The association of an informant report evaluation with a tactile cognitive testing provided high sensitivity to the screening of dementia in older patients.^13^ We did not find tactile memory evaluations compared with batteries based on figures.

The capacity to adequately recollect past events, maintaining their temporal order of occurrence, is usually evaluated as delayed memory recall, also known as autobiographical memory or episodic memory, and is accepted as a marker for AD.^14^ ^,^ ^15^ ^,^ ^16^ A research evaluating tactile recognition of the high-relief-engraved patterns was applied to patients in early stages of dementia, showing that patients with AD made more errors in remembering the correct sequence of stimuli,^17^ leading to the conclusion that delayed recall memory or episodic memory was affected in those patients. To the best of our knowledge, that represents the only study carried out focused on tactile episodic memory in patients with Alzheimer’s dementia. There was no mention in the literature of any studies comparing latter delayed haptic memory and visual memory evaluation. We developed a battery based on tactile perception, recognition, and recollection of everyday objects, testing tactile delayed recall memory discrimination and late recognition. The battery was applied to patients with AD and compared with a validated visual-based cognitive test, i.e., Brief Cognitive Battery – Education (BCB-Edu).^18^ The aim of this study, preceding the validation of the diagnostic sensitivity and specificity data, is to compare the results of haptic memory tests with a validated similarly structured tests based on visual and verbal information. Both were applied to patients with AD.

## METHODS

### Study design

A cross-sectional observational study, with patients as their own controls, was carried out, evaluating patients diagnosed with AD and followed in the Clinics Hospital of Botucatu Medical School in São Paulo, Brazil, over a 6-month period from June to December 2019. The data were collected in the morning. The Research Ethics Committee approved the study and followed the resolution of the Decree of the National Health Council CNS510/2016 related to research with humans. All participants or their legal guardians provided written informed consent. Sixty-four patients were included in this study. The inclusion criteria were probable AD dementia diagnosis by the National Institute on Aging and Alzheimer’s Association (NIA-AA) recommendations,^19^ Clinical Dementia Rating (CDR) between 1 and 2 points,^20^ Hachinski Ischemic Score^21^ ^,^ ^22^ ≤9, and magnetic resonance imaging (MRI) with hippocampal size reduction measured by a trained radiologist. The exclusion criteria were visual or auditory impairment without correction, focal neurological signs, particularly agnosia, and other limiting diseases, such as rheumatoid arthritis and others. The results of our brief screening battery for tactile memory assessment with everyday objects were compared with a brief cognitive screening battery based on pictures — BCB-Edu^18^ and the memory abilities of MMSE.

### Neuropsychological assessment

**Figure 1.**
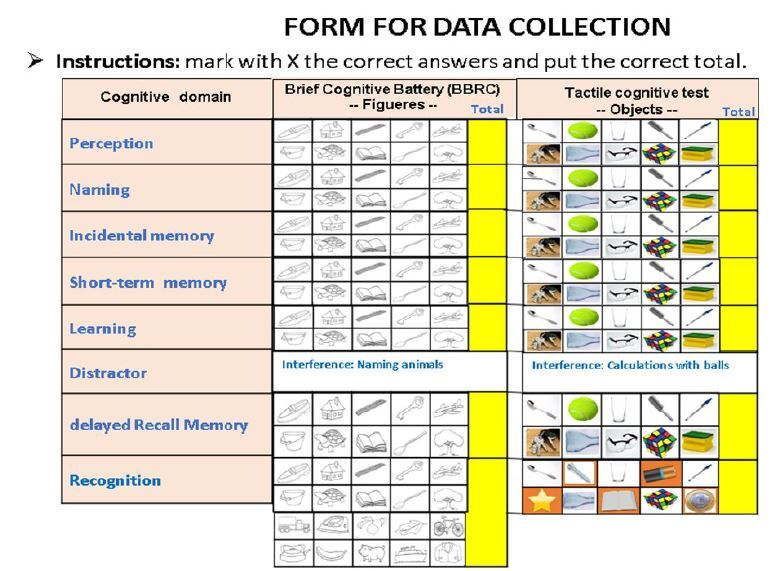
Comparative table of Brief Cognitive Battery and tactile cognitive test used for the data collection: in the left column are the domains evaluated by both tests, in the middle column are the figures used in the battery of Brief Cognitive Battery, in the right column are photos of the objects used for the tactile cognitive test, and the yellow columns are used to put the results of each assessment.

Among other routine examinations, the patients underwent a BCB-Edu test (Figure 1). This battery includes tests of memory and executive function comprising the examinations of visual identification and naming of 10 simple drawings, incidental and immediate memory, learning, verbal fluency, delayed recall (i.e., after 5 min), and recognition tasks (i.e., recognition of 10 familiar figures presented among new ones). A trained neurologist administered the test. Those with compromised episodic memory (i.e., delayed recall with a cut-off score of 7) and affected recognition with CDR between 1 and 2 were classified as having dementia.

**Figure 2.**
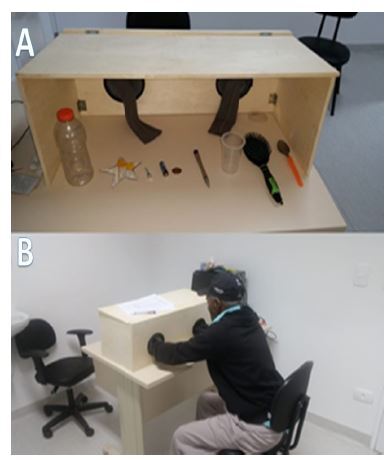
The tactile cognitive test proposed to this study consists in providing standardized everyday objects to visually obliterated patients. The photograph shows two images during the haptic abilities examination. (A) The way in which objects are placed inside a box, out of the patient’s sight. (B) The patient inserting the hands inside the box to be able to manipulate the objects for latter recall.

The tactile cognitive test proposed in this study was applied approximately 20 min later and consists of providing standardized everyday objects to patients who were prevented from seeing the objects (Figures 1 and 2). The test was structured in a similar manner to the validated visual test, as shown in Figure 1, and tested tactile identification and naming, incidental memory (i.e., span), immediate memory (i.e., short-term memory), learning, verbal fluency, delayed recall memory, and recognition. Counting small colored glass balls was used as a distraction before delayed recall and recognition evaluation.

Identification and naming (perception) — the patient was asked to touch with both hands each of 10 everyday objects placed inside a wooden box. They were not able to see the objects but could freely handle them. Initially, each patient was asked to identify and name the familiar objects. The examiner could offer verbal assistance. If the patient was not able to name the object, he/she was asked what the object would be used for.

Incidental memory (attention) — attention was ascertained by asking the patients to select the objects presented in the identification and naming examination.

Immediate memory (short-term memory) — Patients were asked to touch each of the objects again over 5 sec. Thereupon, they were then instructed to name the objects that they had touched, with a maximally tolerable threshold of 60 sec to answer.

Learning test — the 10 objects were made available again for tactile perception (5 sec). The evaluator instructed each patient to try to memorize the objects by their touch and their placement. Shortly after, each patient was asked to name the objects within 60 sec. The number of mistakes and successes were recorded.

Distraction — as a distraction, each patient was asked to pick up a certain number of glass balls placed in a box blocked from view and was directed to report how many they had taken over a period of at least 5 min.

Delayed recall test (after 5 min) — soon after the conclusion of the distraction, the examiner asked the patient again to report the items touched in the previous object section. The patient was considered to have a successful recall if he/she provided the names of the objects like BCB-Edu. Each successful answer received 1 point.

Recognition — five objects were randomly selected along with five new ones. Each patient was asked to report which objects had been felt previously and which ones were new. The number of correct answers was recorded.

### Statistical analysis

Mean, standard deviation, maximum, minimum, and median were calculated for all demographic variables and questionnaire responses. Frequency and percentages were obtained for categorical variables. Pearson’s correlations were applied to compare items on the visual and tactile batteries. The paired Student’s *t*-tests were used to compare between means, taking into account that patients were used as their own controls. The significance level was set at 5% for the data analysis. Analyses were performed on SAS software for Windows v.9.4.

## RESULTS

### Demographic features

Sixty-five patients were initially considered for the study. Of the total sample, 40 had limiting diseases, including 2 with rheumatoid arthritis, 7 visual and or auditory alteration, 1 with chronic obstructive pulmonary disease, and 7 with neurological symptoms such as peripheral neuropathies and agnosia. Therefore, they were not included along with 7 patients who did not consent to participate in the battery of tests. We were thus left with 21 patients who completed the full examination, with mean CDR 1.6 and SD 0.47. Of the total sample, 57.1% were females, the mean age of this sample was 76.2±7.3 years old, and the mean formal education level was 3.1±2.0 years, indicating a low level of schooling. Overall, 47.6% were married (Tables 1 and 2).

**Table 1.** Descriptive analysis for sociodemographic, Mini-Mental State Examination, and Clinical Dementia Rating data.

	Number of patients	Mean	Standard deviation	Minimum	Maximum	Median
Age	21	76.2	7.3	54.0	87.0	77.0
Schooling	21	3.1	2.0	0.0	8.0	3.0
Hachinski Ischemic Score	21	5.5	1.4	3.0	8.0	6.0
CDR	21	1.66	0.48	1.0	2.0	2.0
MMSE	21	12.4	4.5	6.0	20.0	13.0
Incidental memory (verbal) MMSE	21	2.5	0.8	1.0	3.0	3.0
Delayed recall (verbal) MMSE	21	0.2	0.5	0.0	2.0	0.0

MMSE: Mini-Mental State Examination; CDR: Clinical Dementia Rating.

**Table 2.** Descriptive analysis for the data of tactile- and visual (Brief Cognitive Battery – Education)-based batteries.

	Number of patients	Mean	Standard deviation	Minimum	Maximum	Median
Visual identification	21	9.6	0.9	7.0	10.0	10.0
Visual naming	21	9.5	1.1	7.0	10.0	10.0
Visual incidental memory	21	2.1	1.8	0.0	7.0	2.0
Visual immediate memory	21	3.5	2.2	0.0	9.0	4.0
Visual learning test	21	4.4	2.2	2.0	10.0	4.0
Visual delayed recall test	21	2.4	2.6	0.0	9.0	2.0
Visual recognition	21	5.7	3.5	0.0	10.0	5.0
Tactile identification	21	9.7	0.7	8.0	10.0	10.0
Tactile naming	21	9.8	0.6	8.0	10.0	10.0
Tactile incidental memory	21	3.4	1.3	1.0	6.0	3.0
Tactile immediate memory	21	3.8	1.3	2.0	7.0	3.0
Tactile learning test	21	4.9	2.1	1.0	9.0	4.0
Tactile delayed recall test	21	3.1	1.4	0.0	6.0	3.0
Tactile recognition	21	7.5	2.7	1.0	10.0	9.0

BCB-Edu: Brief Cognitive Battery – Education.

### Clinical and neuropsychological features

As shown in Table 3, identification, naming, incidental memory, and delayed recall test had no or low correlation, and the short-term, learning test and recognition show good correlation between the visual and tactile batteries. There was no correlation with the memory tests of MMSE.

**Table 3.** Pearson’s correlations of verbal (Mini-Mental State Examination), visual (Brief Cognitive Battery – Education), and tactile abilities among patients with Alzheimer’s disease.

	Pearson’s correlations								
	Incidental memory (verbal)	Delayed recall (verbal)	Tactile identification	Tactile naming	Tactile incidental memory	Tactile immediate memory	Tactile learning test	Tactile delayed recall test	Tactile recognition
Incidental memory(verbal)	1	0.27309	-0.15223	-0.06608	-0.46725	-0.32995	-0.4351	-0.40512	-0.32544
p-value		0.231	0.5101	0.776	0.0327	0.1441	0.0487	0.0685	0.15
Delayed recall (verbal)	0.27309	1	-0.08919	0.14891	0.16847	-0.14951	-0.2079	-0.08334	0.24863
p-value	0.231		0.7006	0.5194	0.4654	0.5177	0.3657	0.7195	0.2771
Visual identification	0.16468	0.18091	-0.22185	-0.1852	-0.33175	0.19604	0.22621	0.05528	-0.0654
p-value	0.4756	0.4326	0.3338	0.4216	0.1418	0.3944	0.3241	0.8119	0.7782
Visual naming	0.13849	0.18991	-0.23289	-0.19442	-0.32494	0.08254	0.2771	0.05065	0.0985
p-value	0.5494	0.4096	0.3096	0.3984	0.1507	0.7221	0.224	0.8274	0.671
Visual incidental memory	-0.31373	0.0981	0.05066	0.01057	0.38871	0.35743	0.38864	0.17798	0.07645
p-value	0.1661	0.6723	0.8274	0.9637	0.0816	0.1117	0.0817	0.4402	0.7419
Visual immediate memory	-0.55172	-0.09284	0.17595	0.05875	0.31281	0.55278	0.68198	0.51144	0.21093
p-value	0.0095	0.689	0.4455	0.8003	0.1674	0.0094	0.0007	0.0178	0.3587
Visual learning test	-0.56519	0.05821	0.38071	0.37607	0.56181	0.72971	0.7159	0.54075	0.46355
p-value	0.0076	0.8021	0.0886	0.0929	0.008	0.0002	0.0003	0.0114	0.0343
Visual delayed recall test	-0.54245	0.05636	0.41918	0.30972	0.48013	0.56813	0.57425	0.42871	0.48834
p-value	0.0111	0.8083	0.0586	0.1718	0.0276	0.0072	0.0065	0.0525	0.0247
Visual recognition	-0.37353	0.20401	0.38341	0.59998	0.33296	0.36531	0.63642	0.52369	0.72762
p-value	0.0953	0.3751	0.0862	0.004	0.1403	0.1034	0.0019	0.0148	0.0002

BCB-Edu: Brief Cognitive Battery – Education; MMSE: Mini-Mental State Examination.

Table 4 indicates that incidental memory and recognition were significantly greater in the tactile evaluation than in the visual evaluation. The other variables had no significant differences. When comparing both tests with the MMSE that evaluates verbal memory, we can note that the delayed recall test was significantly greater and only the verbal incidental was significant, comparing the verbal and tactile test.

**Table 4.** Paired sample analysis for visual, verbal, and tactile data.

	Visual test (BCB-Edu)		Tactile test		p-value
	Mean	Standard deviation	Mean	Standard deviation	
Identification	9.6	0.9	9.7	0.7	0.7406
Naming	9.5	1.1	9.8	0.6	0.3426
Incidental memory	2.1	1.8	3.4	1.3	0.0019
Immediate memory	3.5	2.2	3.8	1.3	0.5601
Learning test	4.4	2.2	4.9	2.1	0.2336
Delayed recall test	2.4	2.6	3.1	1.4	0.2048
Recognition	5.7	3.5	7.5	2.7	0.0021
	**Verbal test (MMSE)**		**Visual test (BCB-Edu)**		
Incidental memory	2.5	0.8	2.1	1.8	0.3736
Delayed recall test	0.2	0.5	2.4	2.6	0.0009
	**Verbal test (MMSE)**		**Tactile test**		
Incidental memory	2.5	0.8	3.4	1.3	0.0251
Delayed recall test	0.2	0.5	3.1	1.4	<0.0001

BCB-Edu: Brief Cognitive Battery – Education; MMSE: Mini-Mental State Examination.

## DISCUSSION

Almost all routine tests used to determine the short- and long-term (i.e., declarative) memory are based on visual and auditory inputs. Tests based on pictures (i.e., figures) are considered the gold standard in measuring episodic memory in elderly individuals.^23^ It is worth noting that either tactile short-term memory (p=0.0019) as tactile recognition (p=0.0021) were comparable and even greater than the observed correlates in the visual picture test (Table 4). Recognition includes a recollection of past experiences and is a central core in the determination of episodic memory being particularly useful in the early detection of AD.^24^ ^,^ ^25^ In this sense, it was possible to compare tactile delayed recall memory with verbal delayed memory (MMSE) (p<0.0001), to test based on pictures (p=0.0009) (Table 4).

The findings are of great relevance, as we know that haptic memory takes some time to develop in the first months of postnatal life.^7^ This is a result of the extended process of connecting touch with other sense modalities, which provide new kinds of information from birth (e.g., vision and audition).^26^ The development of this system requires more complexity of interconnections as individuals move through adulthood and is not well understood with aging. A reduction in the ability to recall auditory and visual events has been observed in elderly people,^27^ with a reduction in performance at approximately 60 years of age, and further delay in recall in even older individuals.^28^ Whether haptic abilities decrease due to aging, especially compared with visual and auditory capacities, has not been adequately determined. Impairment in 3D visual object discrimination is an early predictor of AD,^29^ and a reduction in tactile angle discrimination in patients with mild cognitive impairment and AD has also been reported.^30^ It is important to point out the cited study in which the participants were asked to touch a high-relief surface that could not be considered as a haptic, 3D assessment. The same comment could be applied to a study performed more than 30 years ago, showing the correlations of delayed recall memory based on a high-relief-engraved stimulus with cognitive deterioration in patients with AD.^17^ The proposed haptic testing suggests that a poorer tactile perception is still associated with a good performance of haptic recognition using visual recognition of 3D objects as a parameter (Table 3), which supports the possibility that haptic examination is a promising method to be applied to AD diagnosis and outcome of dementia. Touch thresholds decline with aging,^31^ as well as spatial acuity,^32^ due to peripheral neurological diseases^33^ and other causes including rheumatologic diseases. This suggests a paradox in that aging causes the deterioration of the tactile sensitivity threshold but, nonetheless, maintains a relative preservation of haptic memory. It is of note that our results demonstrated a good correlation of tactile memory with visual memory for most direct comparisons. Many studies have identified the central role of the perirhinal cortex, either by 3D tactile screening battery or by functional magnetic resonance images, as a key site of tissue loss in AD.^34^ ^,^ ^35^ ^,^ ^36^ The supposition that 3D tactile perception is complemented by mental imaging of target objects may lead to the conclusion that 3D tactile tests are applicable in the clinical diagnosis of AD. This presumption was confirmed by another battery,^11^ which is based on combined visual and tactile stimulus showing good accuracy on detection of memory impairment in demented patients. A good correlation was also achieved comparing the haptic perception and recognition of objects and the suitable place for fitting either to verbal perception or to verbal recognition (MMSE) in demented patients.^10^ There is a dominance of the visual system in the aging brain, which means, with decreasing of vision, it could cause an enhancement of haptic recognition,^37^ preserving the mental remembrance of objects which may occur in aging. The apparent better performance of both tactile incidental memory and recognition among our Alzheimer’s group of patients could be explained by the results of a previous functional MRI (fMRI) experiment on the cross-modal links between vision and touch, detailing that complex haptic texture representations include the visual cortex and association cortices as well as the somatosensory cortex.^38^ This comprehensive representation of tactile connections along brain tissue can explain, at least in part, the perceived persistence of haptic recognition in older disabled patients in the studied sample. There are many questions to be answered. One question is whether haptic abilities are preserved even when visual and auditory capacities are affected in older patients with dementia. The possibility that haptic abilities persist for a longer time in patients with AD should be considered. Studies with age and sex-matched control groups could help to respond to the question of applicability in an early phase of AD versus advanced disease staging. The display of the all-day objects to be touched and identified by the cognitively impaired patients offers an alternative, moreover, when patients show visual deficiencies so common in elderly people. It is also expected to find promising results with test applicability in low-schooling demented patients. Even with the limitations to our conclusions, it could be stated that the tactile examination could offer an alternative to the evaluation of demented patients with good correlation with the usual pictures and verbal-based tests, particularly to the determination of episodic memory impairment. The data from the ongoing study could bring a broader understanding regarding the sensitivity and specificity of the proposed haptic test battery in clinical practice.

This study shows evidence of possible applicability of haptic sensibility examination applied to the diagnosis of AD memory dysfunction.

Tactile recognition may be best preserved in Alzheimer’s demented patients compared with visual recognition.
